# NKD2 Trigger NF-κB Signaling Pathway and Facilitates Thyroid Cancer Cell Proliferation

**DOI:** 10.1007/s12033-023-00665-7

**Published:** 2023-02-23

**Authors:** Shaoying Ke, Qunxiong Pan, Congren Wang, Zijian Su, Mingzhu Li, Xiaoyu Liu

**Affiliations:** Department of Throid Surgery, Quanzhou First Hospital Affiliated to Fujian Medical University, Quanzhou, 362000 Fujian China

**Keywords:** Proliferating, THCA, NF-κB, TGCA, GSEA

## Abstract

NKD inhibitor of WNT signaling pathway 2 (NKD2) is an emerging player in cancer onset and progression. Here, it was confirmed that THCA patients have robustly expressed NKD2, which was linked to an advanced pathologic stage. The prognosis was worse for those with high NKD2 levels. Functionally, ectopically produced NKD2 promotes THCA cell proliferation, whereas NKD2 knockdown impairs the ability of THCA cells to proliferate. Mechanically, ectopically expressed NKD2 activated NF-κB transcriptional activity, whereas NKD2-deficient THCA cells showed lower NF-κB transcriptional activity. As a result, NKD2 activates the NF-κB signaling pathway, encouraging the growth of THCA cells.

## Introduction

Thyroid cancer (THCA) is the most common cancer of the endocrine system. Cancer statistics in 2022 demonstrate that THCA occurs in 43,800 patients and is responsible for 2230 cancer-associated death [1]. The incidence of THCA has declined in recent years as a result of therapeutic practices intended to reduce over-detection. Thyroidectomy and radioactive iodine treatment benefit cancer patients [2]. However, advanced THCA patients have a poor 5-year survival rate [3]. Therefore, uncovering the mechanism behind THCA development is critical to exploring novel therapeutic targets against THCA.

NKD inhibitor of WNT signaling pathway 2 (NKD2) is a member of the Naked family which functions as a negative regulator of Wnt receptor signaling through interaction with Dishevelled family members [4]. The encoded protein participates in the delivery of transforming growth factor alpha-containing vesicles to the cell membrane [5]. Its involvement in the onset and development of different cancers was described [6]. For instance, highly expressed *NKD2* in ovarian cancer constrain cancer cell growth and survival [7]. *NKD2* DNA methylation lessens the invasion and migration of breast cancer cells [8]. Low expression of NKD2 is also associated with hepatocellular carcinoma prognosis [9]. However, its bio-function in THCA remains unknown. Therefore, we first assessed the NKD2 expression in pan-cancers and THCA and delineated the clinical significance in THCA, and analyzed the function of NKD2 during THCA malignancy by both gain and loss of functional assays. Finally, we uncovered the underlying mechanism behind the NKD2-mediated action in THCA progression.

## Methods

### Gene Expression Datasets

Based on the TCGA database [10] (https://tcgadata.nci.nih.gov/tcga/) and Genotype-Tissue Expression GETx database [11] (https://commonfund.nih.gov/GETx/), the expression profile of NKD2 in pan-cancer and THCA was examined using the R package TCGA biolinks (accessed on 22 June 2022). The receiver operating characteristic (ROC) curve was also established Using “plotROC” in R software [12]. The GEO database [13] (GSE33630, GSE3467, and GSE3678) was also retrieved to verify the NKD2 differential expression in THCA tissues and normal tissues. Based on data from TCGA-THCA, univariate analysis was also used to verify the association of *NKD2* expression with the clinicopathological characteristics of THCA patients. R software with survival and survminer packages [14] (R software version 3.3) was applied to determine the prognostic significance of NKD2 in THCA. The ggplot2 and heatmap package in R software (R software version 3.3) [15] was applied to visualize the differential expression in the high- and low-expressed *NKD2* events. Signaling pathway impact analysis (SPIA) R package (v3.3.3) (R software version 3.3) [16] was employed to enrich the pathways of the differentially expressed mRNAs in *NKD2*-high and *NKD2*-low groups according to the median value of NKD2 expression.

### Cell Culture and Transfection

Human papillary thyroid cancer cell lines TPC-1 and K1 were commercially obtained from the Wuhan university cell bank (Wuhan, China) and maintained in a 5% CO2 atmosphere at 37 °C. RPMI-1640 (EK-bioscience, Wuhan) is required for TPC-1 cells and DMEM (EK-bioscience, Wuhan) for K1 cells.

To enforce NKD2 expression in THCA cells, MYC-tagged *NKD2* overexpression plasmids were purchased from Genechem, China. The MYC-tagged NKD2 plasmids and the empty plasmids were delivered into 2 × 10 [3] PTC-1 and K1 cells using Lipofectamine 2000. 48 h post-transfection, western blot was employed to verify the overexpression of NKD2 in TPC-1 and K1 cells.

CRISPR–Cas9 methods were employed to deplete *NKD2* in PTC and K1 cells. In short, the sgRNAs targeting *NKD2* exon 1 (sgRNA1, AGAGCGTGAACGTCCACTCC) and exon 2(sgRNA2, GGAGCGCAGAAACCACTACC) were designed on http://portals.broadinstitute.org/gpp/public/analysis-tools/sgrna-design. The synthesized oligos were linked into lentiCRISPRv2 and then amplified in 293 T cells by calcium phosphate transfection. 48 h later, the lentiviral particles were collected and filtered before introduction into PTC-1 and K1 cells. The successfully transfected PTC-1 and K1 cells were subjected to antibiotic selection (puromycin). 14 days later, the puromycin-resistant cells were collected for western blot analysis.

### RT-qPCR

Total RNA from TPC-1 and K1 cells was extracted using the Total RNA Extraction Reagent TRIeasy1 kit (Yeasen, China). cDNA was synthesized using PrimeScript RT reagent Kit (Takara, Japan). Following, RT-qPCR amplification reactions were undertaken using M-MLV-RT (Promega, USA) on an ABI 7900HT (Applied Biosystem, USA). Data were processed using the 2^−ΔΔCT^ method [17] with normalization to *GAPDH*.

### Western Blots

5 × 10 [5] TPC-1 and K1 cells were treated with RIPA buffer (Sigma, USA). The supernatant was collected for protein concentrations using the BCA kit (ThermoFisher, USA). Afterward, 20 μg protein sample was loaded on 10% SDS-PAGE. On completion of the electrophoresis, the protein was transferred onto PVDF membranes using the traditional sandwich method. The processed membranes were maintained in a blocking solution (Roche, USA) at room temperature for 30 min before exposure to antibodies at 4 °C overnight. The antibodies were anti-MYC antibody (Cat#sc-40, Santa Cruz Biotechnology, CA), anti-NKD2 antibody (Cat#K110699P, Solarbio, China), anti-GAPDH antibody (Cat#K200057M, Solarbio, China), anti-IκBA antibody (Cat#K101551P, Solarbio, China), anti-p-p65 antibody (Cat#BC336, Affinity Biosciences, China). Consecutive exposure to secondary antibodies (Solarbio, China) was conducted at room temperature for 1 h. Blots were developed using an ECL kit (Amersham, USA). GAPDH was used for normalization.

### CCK8 Assays

5 × 10 [3] TPC-1 and K1 cells were plated on 96-well plates for the next 24 h, 48 h, and 72 h maintenance. 10uL CCK8 reagent was added to each well. 2 h later, the OD450 was read using a plate reader (Biotech, USA).

### Colony Formation Assays

1 × 10 [3] TPC-1 and K1 cells were grown on 6-well plates. 12 days later, the THCA cells were washed with treated with a crystal violet cell colony staining kit (Genemed, China). In the colony, more than 500 cells were counted. Each experiment was in triplicate.

### Statistical Analysis

All data were processed using Graphic prism 9.0 and expressed as mean ± standard deviation (SD). The unpaired student's t-test and one-way ANOVA were applied to analyze the data from two groups or multiple groups. Differences were considered statistically significant at *p* ≤ 0.05.

## Results

### Amplification of NKD2 Correlates with Inferior Prognosis of THCA Patients

**Fig. 1:**
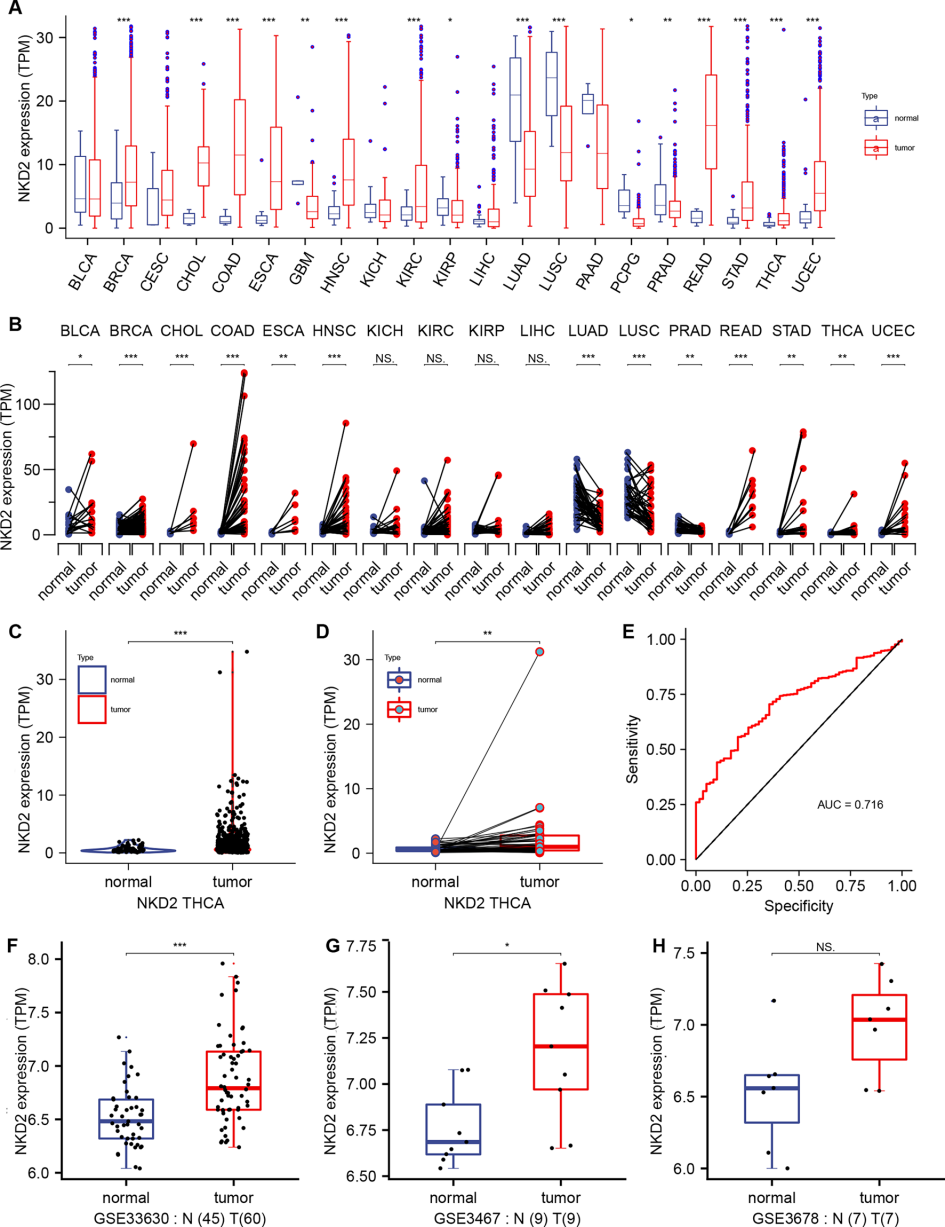
Highly expressed NKD2 in THCA patients. **A** NKD2 expression profile in pan-cancer from the TCGA and GETx databases. Data are shown as the mean ± SD. ***P < 0.001, **P < 0.01, *P < 0.05. **B** *NKD2* expression profile in pan-cancer from the TCGA database. Data are shown as the mean ± SD. ***P < 0.001, **P < 0.01, *P < 0.05. **C** NKD2 mRNA expression in TCGA-THCA tumor tissues in comparison with TCGA normal tissues. Data are shown as the mean ± SD. ***P < 0.001. **D** *NKD2* mRNA expression in TCGA-THCA tissues in comparison with tumor-free tissues from TCGA and GETx. **E** ROC analysis of NKD2 for predicting THCA in TCGA-THCA cohorts. **F** Expression analysis of NKD2 in clinical TGCA samples using public GEO data GSE33630. **G** Expression analysis of NKD2 in clinical TGCA samples using public GEO data GSE3467. Data are shown as the mean ± SD. *P < 0.05. **H** Expression analysis of NKD2 in clinical TGCA samples using public GEO data GSE3678. Data are shown as the mean ± SD. *P < 0.05

**Fig. 2:**
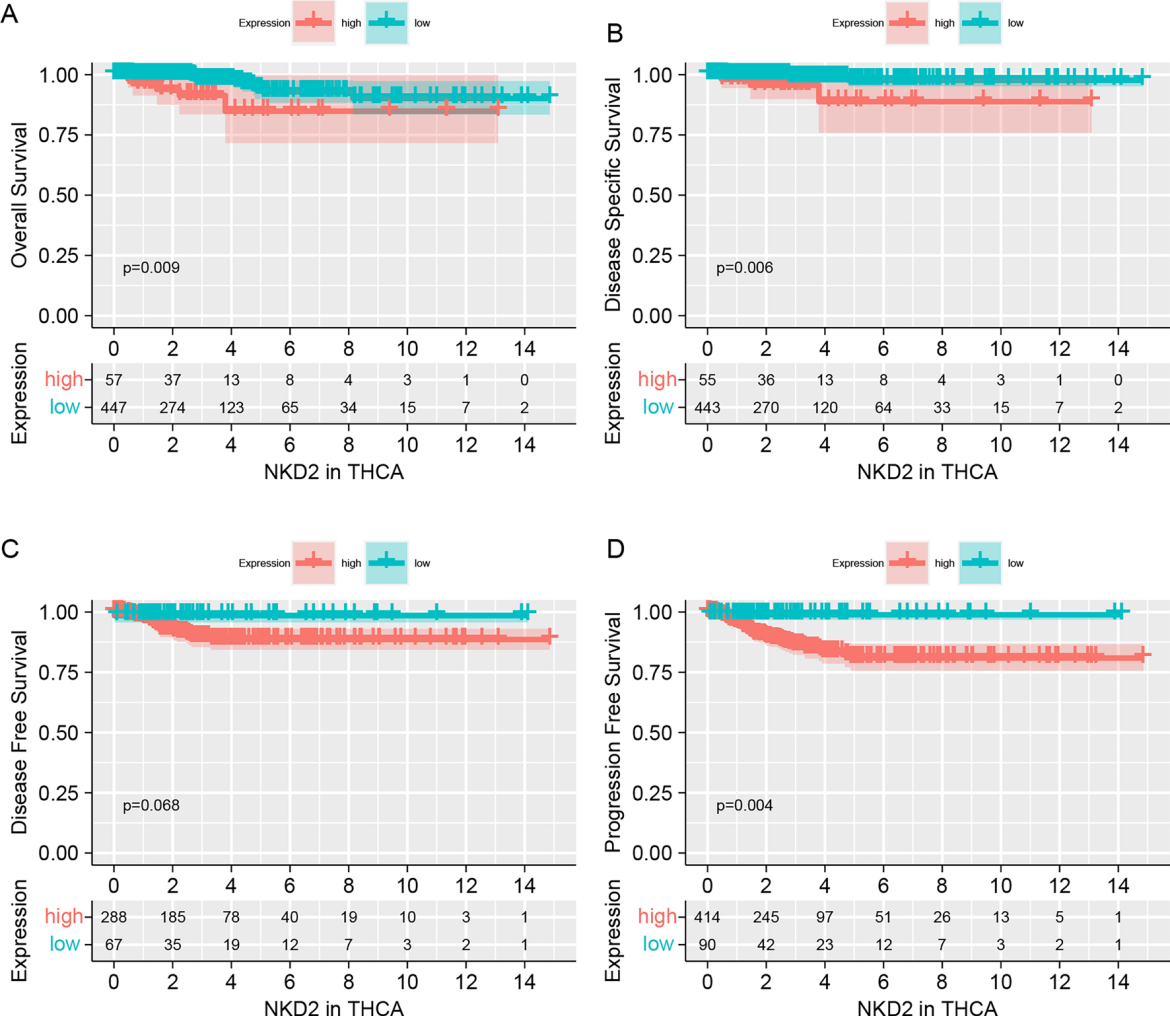
Clinical relevance of *NKD2* with the prognosis of THCA patients. Using median value as a cutoff, TCGA-THCA patients were subgrouped into NKD2-low and *NKD2*-high groups. R2 Genomics Analysis and Visualization Platform were applied to compare the overall survival **A** Disease-specific survival **B** disease-free survival **C** and progression-free survival **D** in *NKD2*-low and *NKD2*-high groups

Given the involvement of NKD2 in carcinogenesis and progression [3, 8], we queried its expression in pan-cancer based using the clinical database from the Cancer Genome Atlas (TCGA) and GETx. *NKD2* mRNA levels were readily detectable in tumor tissues derived from BLCA (Bladder Urothelial Carcinoma), BRCA(Breast invasive carcinoma), CHOL(Cholangiocarcinoma), COAD (Colon adenocarcinoma), ESCA (Esophageal carcinoma), GBM (Glioblastoma multiforme), HNSC (Head and Neck squamous cell carcinoma), KIRP (Kidney renal papillary cell carcinoma), LIHC (Liver hepatocellular carcinoma), LUAD (Lung adenocarcinoma), LUSC (Lung squamous cell carcinoma), PCPG (Pheochromocytoma and Paraganglioma), PRAD (Prostate adenocarcinoma), READ (Rectum adenocarcinoma), STAD (Stomach adenocarcinoma), THCA (Thyroid carcinoma), UCEC (Uterine Corpus Endometrial Carcinoma), and while NKD2 mRNA levels were rarely detected in the corresponding TCGA and GETx normal tissues (Fig. 1A). Consistently, in the TCGA tumor data and paired non-tumor data, we found that the *NKD2* mRNA levels were also robustly expressed in the tumor tissues from BLCA, BRCA, CHOL, COAD, ESCA, GBM, HNSC, KIRP, LIHC, LUAD, LUSC, PCPG, PRAD, READ, STAD, THCA, UCEC compared with the paired tumor-free tissues (Fig. 1B). In THCA, there was an overwhelming enhancement of the NKD2 expression in tumor tissues compared with the tumor-free tissues (Figs. 1C and D). More interestingly, the area under the curve (AUC) showed that NKD2 expression had a higher diagnostic accuracy (AUC = 0.716) (Fig. 1E). Also, we validated the differential expression of *NKD2* in THCA using the GEO database, GSE33630, GSE3467, and GSE3678. Undoubtedly, we also found that NKD2 was expressed at higher levels in THCA tissues relative to that in tumor-free tissues (Fig. 1F–H). Afterward, we analyzed the prognostic significance of NKD2 in the TCGA-THCA cohort. As shown in Fig. 2A, a close correlation between NKD2 mRNA amplification and a low overall survival rate was shown (*p* = 0.009). Lower disease-specific survival rates were seen in TCHA patients with highly elevated NKD2 (*p* = 0.006). The NKD2-high patients had a little poorer survival rate in terms of the disease-free survival of THCA patients (Fig. 2 C, *p* = 0.068). The poor progression of free survival was similarly correlated with the strongly expressed NKD2 (*p* = 0.004, Fig. 2D).

**Fig. 3:**
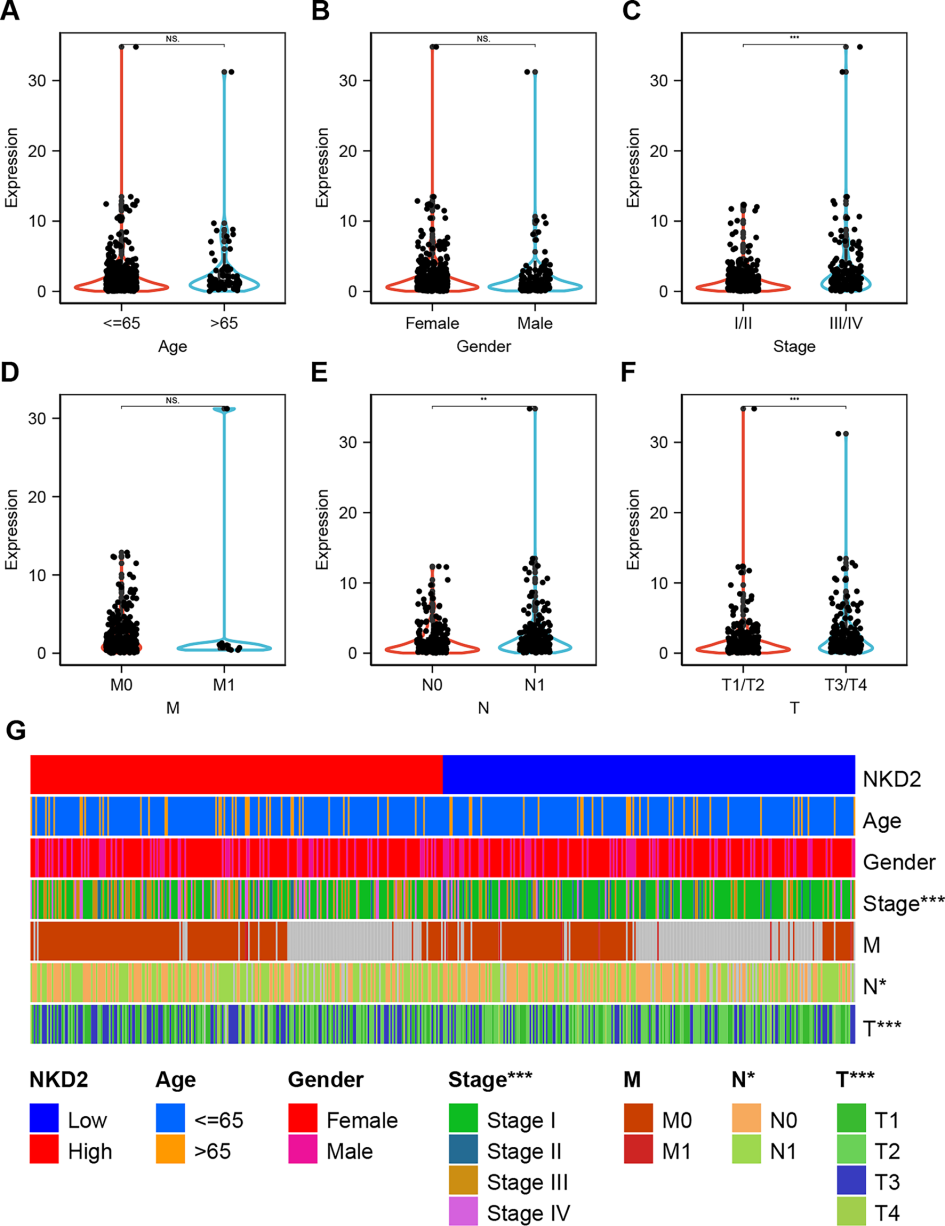
Association of *NKD2* expression with clinicopathological factors in TCGA-THCA patients. The patients in the TCGA-THCA cohort were categorized into ≥ 65 years, < 65 years **A** Female or male **B** I/II and III/IV **C** M0 and M1 **D** N0 and N1 (**E**), T1/T2 and T3/ T4 **F**. Univariate analysis was performed through R (version 3.4.0) to determine the correlation of NKD2 with the above-mentioned factors. **G** Univariate analysis through R summarized the correlation of *NKD2* with age, sex, advanced stage, M stage, nodal status (N), and T stage (T). T (stage): the size of the primary tumor. N (nodes): Whether there is lymph node metastasis. M (metastasis): Whether there is distant metastasis. ***P < 0.001, **P < 0.01, *P < 0.05

Additionally, we also downloaded the clinical data of the TCGA-THCA cohort and analyze whether NKD2 expression is related to the clinicopathological characteristics of THCA patients. As shown in Fig. 3, In the TCGA-THCA cohort, NKD2 expression was amplified in patients with advanced T(tumor) (T3/T4) and N(Node) (N1) stages. Taken together, these data strongly imply that NKD is involved in thyroid carcinogenesis and progression.

### Analysis of NKD2-Associated Signaling Pathway

**Fig. 4:**
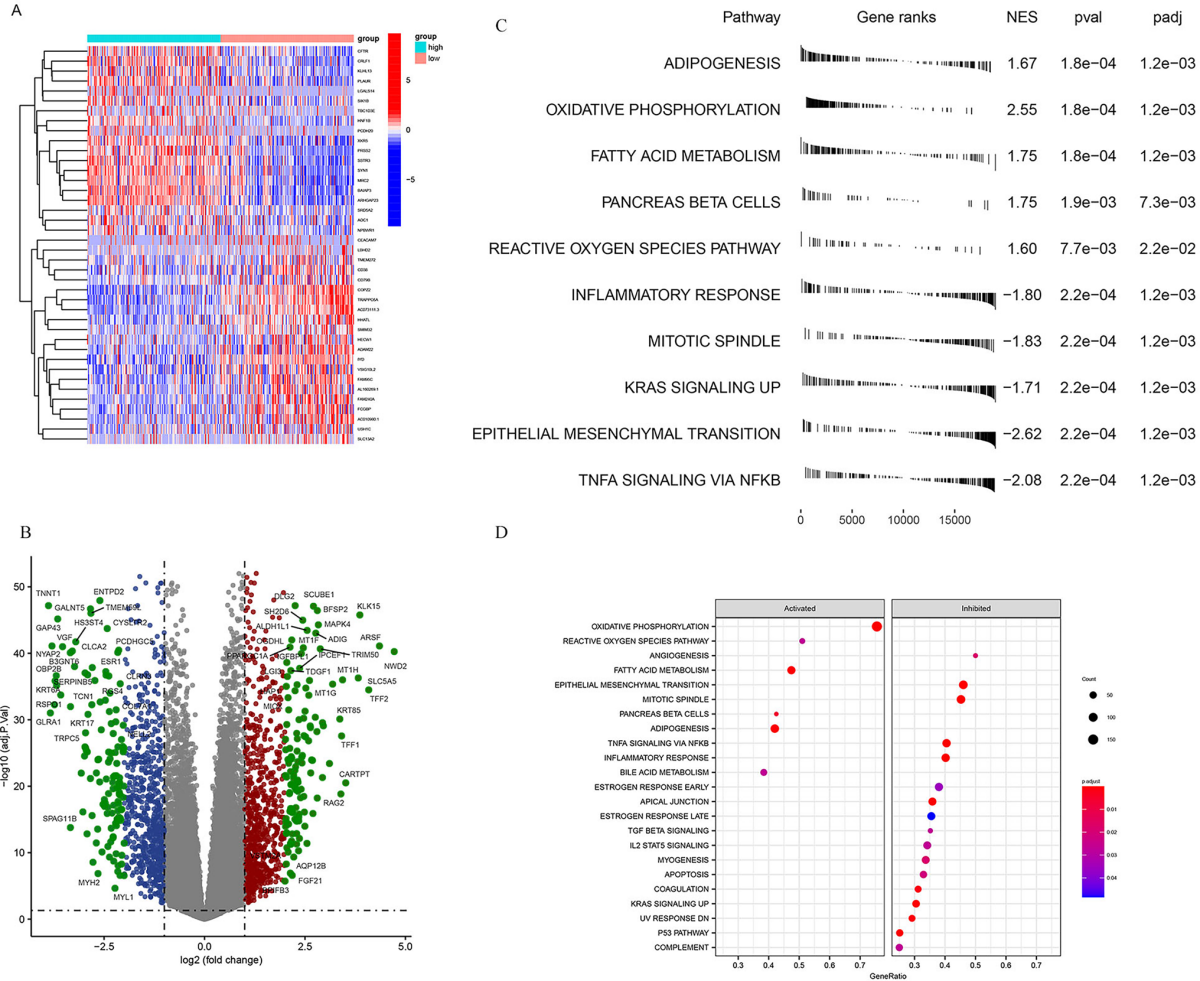
Functional enrichment of high-*NKD2* and low-*NKD2* THCA patients. **A** Heatmaps visualizing differential expressed genes between high-*NKD2* and low-*NKD2* patients. **B** Volcano plot showing differential expressed genes between high-*NKD2* and low-*NKD2* patients. **C** Gene set enrichment analysis (GSEA) of the gene expression changes in high-*NKD2* and low-*NKD2* patients. **D** plot of GSEA KEGG pathway analysis

To decipher the mechanism behind the pro-oncogenic role of NKD2 during THCA progression, we first found the differentially expressed genes in *NKD2*-low and *NKD*2-high groups based on the TCGA-THCA cohort (Fig. 4A and B). Further GSEA analysis was undertaken to annotate the regulatory pathways by NKD2 (Fig. 4C). Among them, the differently expressed genes were mostly enriched in adipogenesis (NTES = 1.67) oxidative phosphorylation (normalized enrichment scores, NES = 2.55), fatty acid metabolism (NES = 1.75), pancreas beta cells (NES = 1.75), reactive oxygen species pathway (NES = 1.60), inflammatory response (NES =  − 1.80), mitotic spindle (NES = 1.83), KRAS signaling up (NES =  − 173), epithelial-mesenchymal transition (NES =  − 2.62), and TNF-α signaling via NF-κB (NES =  − 2.08). Consistently, Gene Ontology (GO) signaling pathway enrichment analysis (Fig. 4D) demonstrated the enrichment in Oxidative phosphorylation, reactive oxygen species pathway, angiogenesis, fatty acid metabolism, epithelial-mesenchymal transition, mitotic spindle, pancreas beta cells, adipogenesis. Especially, the *NKD2* downregulation suppressed TNF-α signaling via NF-κB. Therefore, we reasoned that the TNF-α/NF-κB signaling pathway is required for NKD2 influence on the THCA progression.

### NKD2 Overexpression Enhances THCA Cell Proliferation

**Fig. 5:**
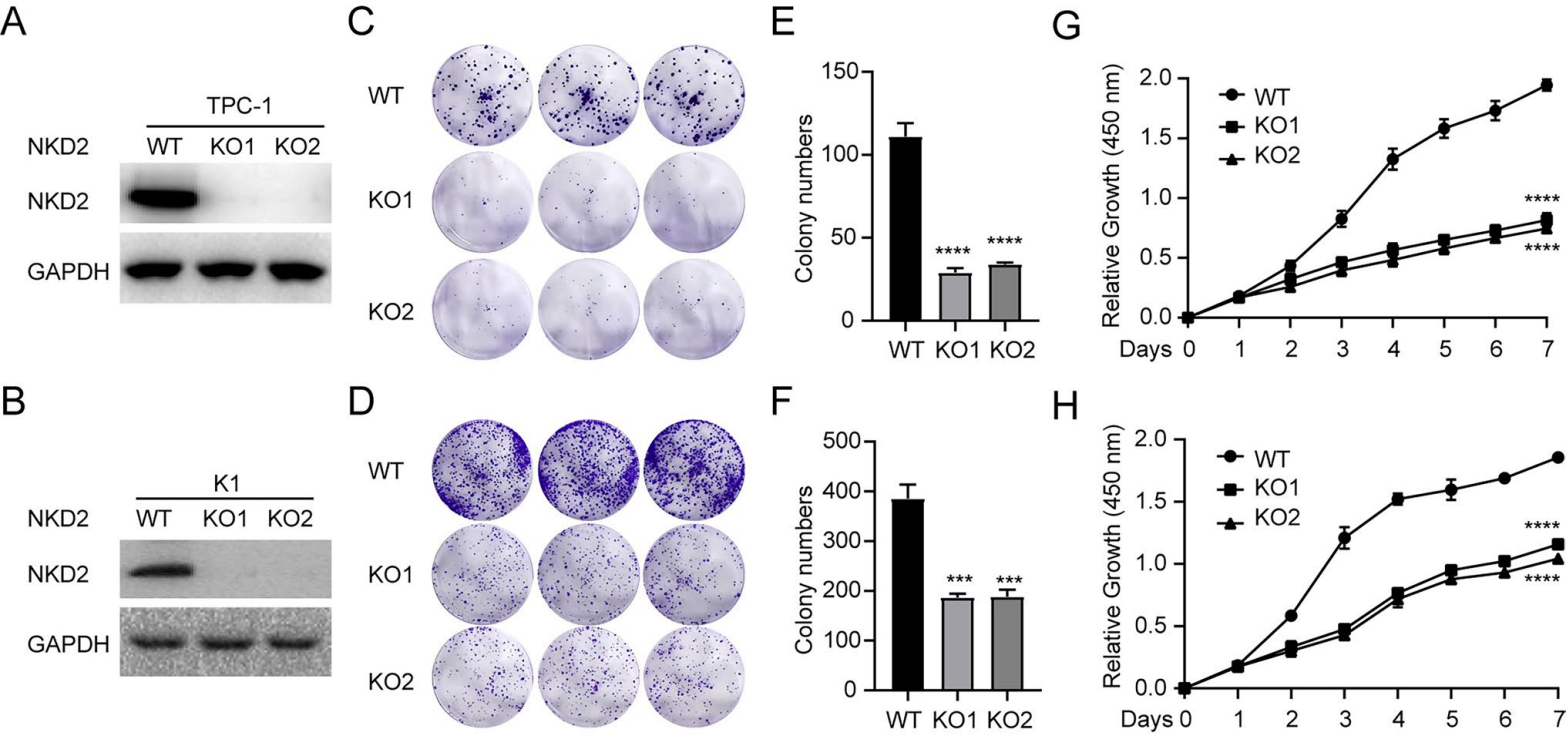
NKD2 overexpression enhances THCA cell proliferation. TPC-1 and K1 cells were transfected with pIRES-Myc constructs or pIRES-NKD2-Myc constructs. 48 h later. **A-B** NKD2 expression was analyzed by Western blot. **C-F** Colony formation assays assessing the THCA cell colony formatting rate after NKD2 overexpression. **G-H** CCK8 assays quantifying the THCA cell proliferation after NKD2 overexpression. ***P < 0.001, ****P < 0.0001

The clinical importance of NKD2 in THCA progression promoted us to verify the function of NKD2 in vitro. To do it, we enforced the NKD2 expression in two THCA cells, TPC-1 and K1 cells (Fig. 5A and B). Expectedly, along with the NKD2 overexpression, both THCA cells presented a higher colony formatting rate (Fig. 5 C–F). Consistently, NKD2 overexpressing led to an elevation in the proliferation of TPC-1 and K1 cells (Fig. 4G–H). These data supported a promoting function of NKD2 during THCA malignancy.

### NKD2 Depletion in THCA Cells Diminishes Cell Proliferation

**Fig. 6:**
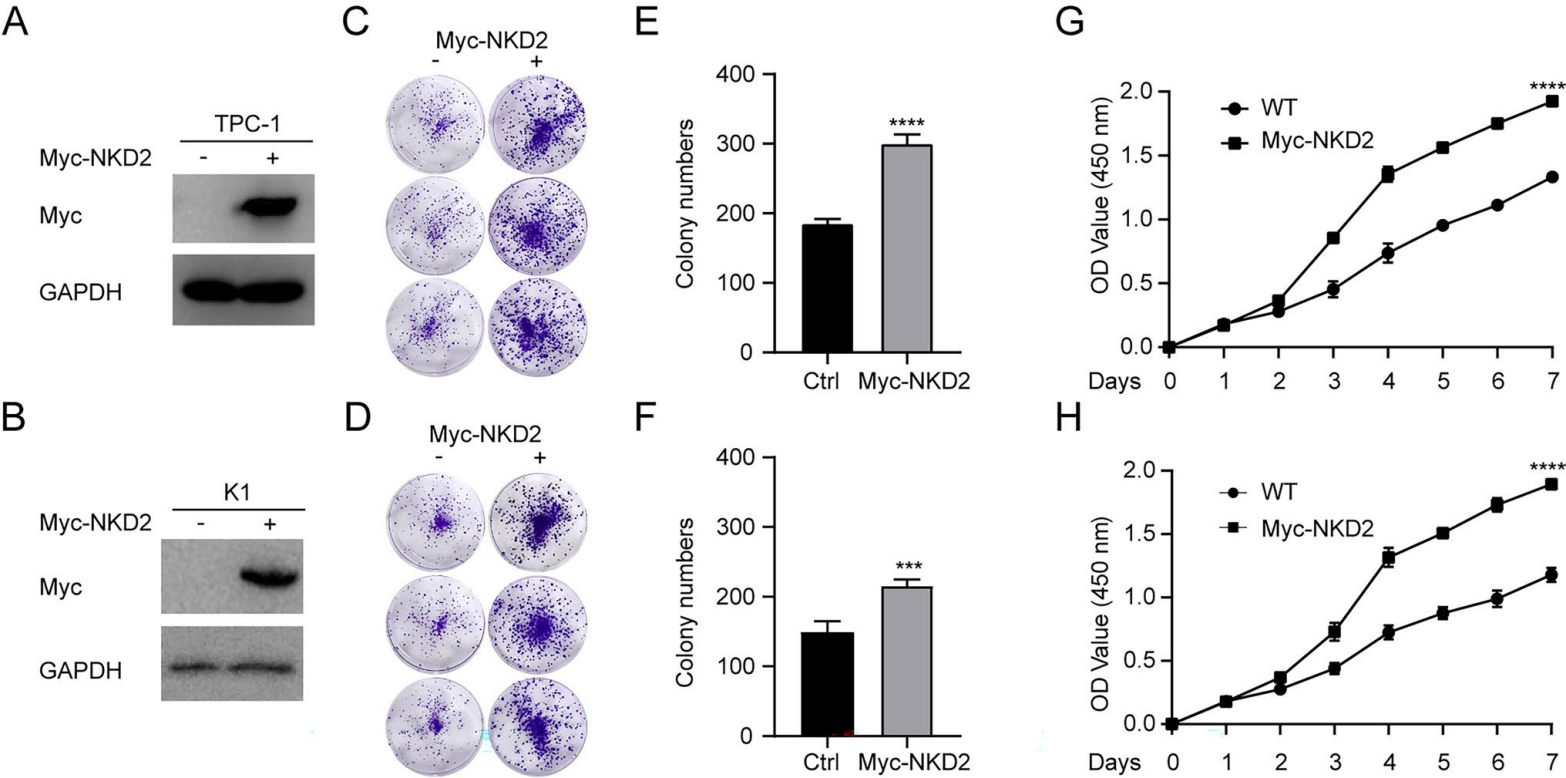
NKD2 depletion in THCA cells diminishes cell proliferation. After CRISPR/Cas9-mediated NKD2 depletion was conducted in TPC-1 and K1 cells. **A-B** Western blot examining the NKD2 expression. **C-F** Colony formation assays assessing the THCA cell colony formatting rate after NKD2 depletion. **G-H** CCK8 assays quantifying the THCA cell proliferation after NKD2 depletion. ***P < 0.001, ****P < 0.0001

In the interest of ensuring the function of NKD2 during THCA malignancy, CRISPR/Cas9-mediated NKD2 depletion was conducted. When NKD2 knockout (Fig. 6A and B), the proliferative phenotypes of THCA cells were examined. As illustrated in Fig. 6C and F, NKD2 depletion contributed to the decreased colony formation capacity of THCA cells. In parallel, the reduction of THCA cell proliferation was observed using CCK8 assays upon NKD2 depletion (Fig. 6G and H). Collectively, these outcomes suggested that the observed reduction in THCA cell proliferation resulted from NKD2 depletion.

### NKD2 Consistently Promotes the NF-κB Signaling Pathway

**Fig. 7:**
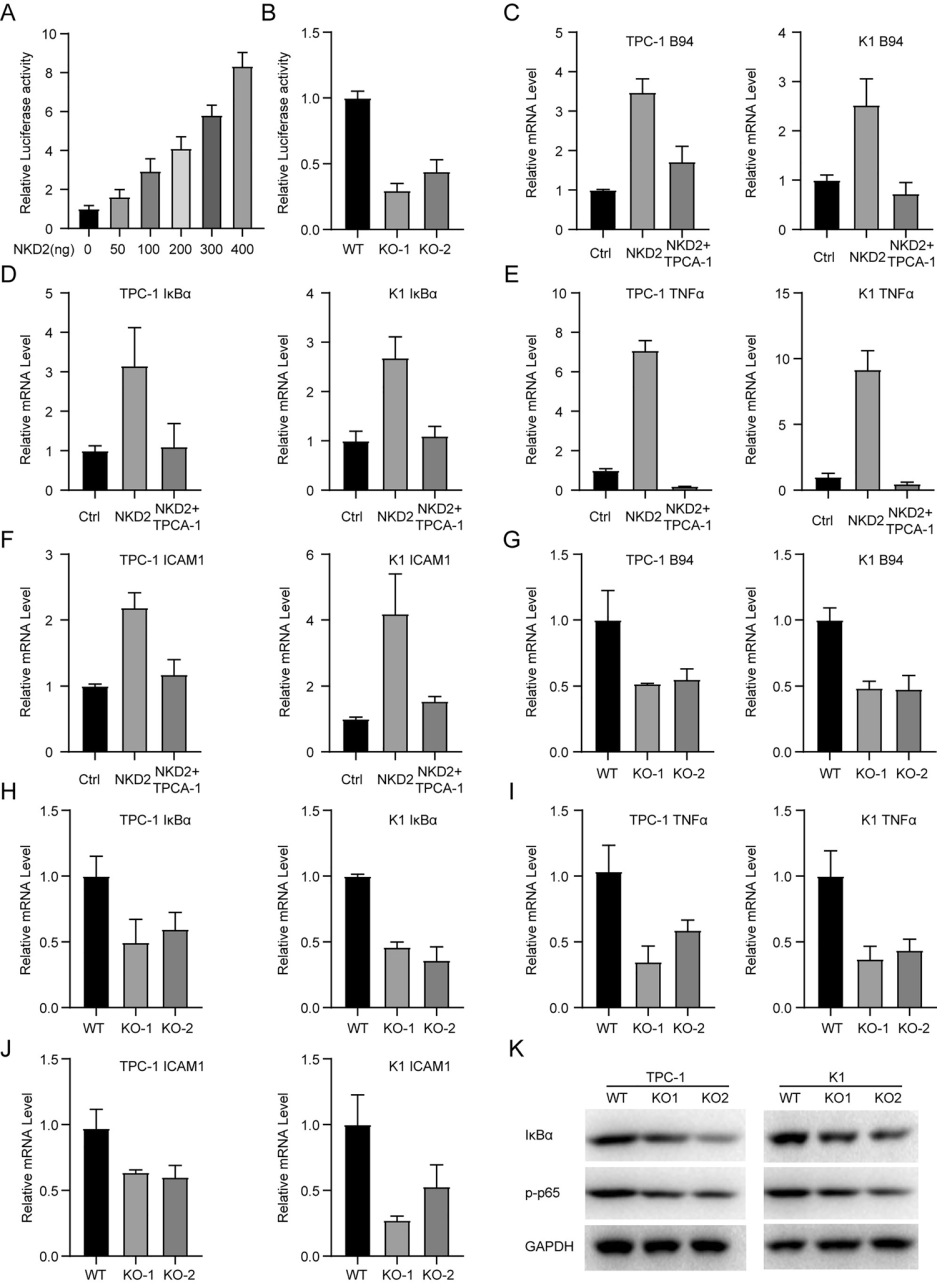
NKD2 consistently promotes the NF-κB signaling pathway. **A** NF-κB luciferase reporter plasmids with IRES-NKD2 constructs (0, 50, 100, 200, 300, 400 ng) were delivered into TPC-1 cells. 48 h later, the luciferase activity was assessed. **B** NF-κB luciferase reporter plasmids were delivered into NKD2-deficient K1 cells. 48 h later, the luciferase activity was assessed. **C**–**G**. NKD2 overexpressing TPC-1 and K1 cells when subjected to TPCA-1 treatment or not. The normal TPC-1 or K1 cells are the control cells. RT-qPCR analyses were conducted to assess the expression of B94 (**C**), IκBa (**D**), TNF-α (**E**), and ICAM1 (**F**). G-J.Two NKD2-deficient THCA cells were subjected to RT-qPCR analysis. The expression levels of B94 (**G**), IκBa (**H**), TNF-α (**I**), and ICAM1 (**J**). **K** Western blots assessing the expression of IκBa and p-p65 in Two NKD2-deficient THCA cells. The untransfected cells were control cells. GAPDH was used for normalization

To support this finding, we co-transfected NF-κB luciferase reporter plasmids with IRES-NKD2 constructs (0, 50, 100, 200, 300, 400 ng) into TPC-1 cells. As a result of NKD2 overexpression in THCA cells, the NF-κB luciferase activities were increased gradually with the dose of NKD2 expression levels (Fig. 7A). And yet, the downregulation of NF-κB luciferase activities was observed in the NKD2-deficient TPC-1 cells (Fig. 7B). Prior work found that B94 (TNF alpha-induced protein 2) [18] and IκBa are the critical part of e TNFA/NF-κB signaling cascade [19]. TPCA-1 is an inhibitor of IKK2, which allow the nuclear translocation of NF-κB subunits and caused NF-κB hyperactivation [20]. Therefore, we assessed the expression of B94, IkBa, and TNF-a in the NKD2 overexpressing THCA cells when TPCA-1 was added or not. Our outcomes from RT-qPCR illustrated an increase in the B94 mRNA levels in TPC-1 and K1 cells when NKD2 was enforced expression, and yet additional treatment of TPCA-1 offset the accumulation of B94 mRNA levels (Fig. 7C). Meanwhile, the strong upregulation of IκBa seen in NKD2-overexpressing THCA cells was deleted when TPCA-1 was supplemented (Fig. 7D). Along with the NKD2 overexpression, a concomitant accumulation of TNF-α expression was also detected in TPC-α and K1 cells, and while an obvious abrogation of the unregulated TNF-α expression occurred in the precedence of TPCA-1 (Fig. 7E). Previous evidence has presented that hyperactivation of the NF-κB signaling pathway causes the expression of intercellular adhesion molecule 1 (ICAM1) and increases cell-endothelial cell adhesion and metastasis, all of which are the hallmarks of a tumor. In THCA cells, NKD2 overexpressing increased ICAM1 mRNA levels, which was abrogated by the post-treatment with TPCA-1 (Fig. 7F). The congruent outcome of RT-qPCR demonstrated that NKD2 depletion in THCA cells decreased the expression of B94, IκBa, TNF-α, and ICAM1 (Fig. 7 I and J). Western blot analysis also confirmed that NKD2 depletion reduced the IkBa expression and lessened the hypophosphorylated p65 (Fig. 7K). Thus, the tumor promotion imposed by NKD2 might be through TNF-α/NF-κB signaling pathway.

## Discussion

Herein, the TCGA-THCA database coupled with the GETx database demonstrated that NKD2 mRNA was amplified in THCA, and patients with highly expressed NKD2 have a more malignant clinical phenotype, including an inferior prognosis. Furthermore, NKD2 promotes THCA cell proliferation by TNF-α/NF-κB signaling pathway.

The tumor-suppressive function of NKD2 is reported in different cancers, due to its inhibition of the WNT signaling pathway [21]. In osteosarcoma, NKD2 decreases the proliferative and metastatic properties [22]. In colorectal carcinoma, downregulating NKD2 endows an oncogenic function [23]. In acute myeloid leukemia, the epigenetic dysregulation of NKD2 is correlated with the dismal clinical outcome with patients [24]. However, its role in THCA remains unknown. Based on the TCGA database, we found the amplification of NKD2 in pan-cancer including THCA. Analyzing the clinical significance of NKD2 showed that NKD2 upregulation was associated with the clinicopathological characteristic of THCA patients and is a favorable prognosis of THCA patients. The clinical significance of NKD2 indicates the essential biofunction during THCA malignancy. Contrary to the previous report, loss and gain functional assays illustrated that NKD2 promotes THCA cell proliferation. Therefore, NKD2 might have dual functions in different cancers.

As an inhibitor of the Wnt signaling pathway, NKD2 is suggested to regulate the wnt signaling pathway in cancers [25, 26]. However, GSEA of THCA cases from TCGA demonstrates that NKD2 is associated with TNFA signaling via NF-κB. Our luciferase reporter assays indicated that NKD2 overexpression triggered the NF-κB-driven transcriptional activity, while the shortened activity was detected in the NKD2-deficient THCA cells. B94, a direct target of TNF-α, is aberrantly expressed in human cancers and could promote cell proliferation, angiogenesis, and metastasis in tumor cells [18]. ICAM1 is a cell surface glycoprotein expressed on endothelial cells and cells of the immune system [27]. It contains NF-κB bind sites and thereby exerts its multiply functions [28]. IκBa is also a critical effector of the NF-κB signaling pathway [19]. The main NF-κB subunit involved in transactivation is p65, and phosphorylation is necessary for p65 to activate transcription [29]. Therefore, we detected the transcription of IκBa, B94, ICAM1, and TNFa in the NKD2-overexpressing THCA cells in the presence of TPCA-1 or not. Our data demonstrated that ectopically expressed NKD2 increased the expression of IκBa, B94, ICAM1, and TNF-α, while this promotion was attenuated by TPCA-1. In parallel, IκBa, B94, ICAM1, and TNFa were also downregulated in NKD2-deficient THCA cells. Our findings for the first time suggested the promoting role of NKD2 in cancer by the TNFA/NF-κB signaling pathway.

We show that NKD2 was unregulated in THCA and an independent risk factor in THCA. Mechanically, NKD2 can trigger the TNFa/NF-κB signaling pathway and promotes THCA cell proliferation. Therefore, our findings for the first time suggest that NKD2 serves as a promoter during THCA malignancy. However, experimental data in vivo should be conducted to validate the findings in vitro. Furthermore, a deeper discovery of the mechanism behind NKD2-driven THCA progression is also required.

## Data Availability

All data were available in the MS.
